# Exploring Resilience and Empowerment in Adults with Reduced Mobility

**DOI:** 10.3390/healthcare13172161

**Published:** 2025-08-29

**Authors:** Raquel Suriá-Martínez

**Affiliations:** Department of Comunication and Social Psychology, Universidad de Alicante, 03690 Alicante, Spain; raquel.suria@ua.es

**Keywords:** resilient capacity, empowerment, functional divergence, training programs

## Abstract

**Background:** In the present study, we examine whether any specific combinations of resilient factors exist that give rise to different resilience profiles in people with reduced mobility. **Methods**: We also verified whether there were any statistically significant differences between the groups obtained regarding empowerment dimensions. The “Resilience Scale” (1993) and the “Empowerment Scale” (1989) were administered to 94 people with reduced mobility aged between 21 and 62 years (*M* = 29.35; *SD* = 6.43). **Results**: The cluster analysis identified three resilient profiles: a low-resilience profile, a group in which Acceptance of Self and Life predominated, and a last group in which high resilience was found across the board. Moreover, statistically significant differences in the empowerment dimensions were observed between the profiles obtained. **Conclusions**: The study findings suggest the need to further explore the subject of resilience as well as the design of programs directed towards enhancing the capabilities of people with disabilities.

## 1. Introduction

In recent years, there has been mounting interest in improving the psychosocial integration of people with *reduced mobility* [1,2,3]. One factor, in particular, has gained relevance in intervention programs to improve the group’s psychosocial integration, strength and quality of life generally, and that factor is empowerment [4].

### 1.1. The Role of Empowerment and Reduced Mobility

While “empowerment” can mean to give power, it commonly refers to a person’s strengthening of capabilities [4]. The concept thus refers to each person’s potential to achieve self-defined objectives and goals, approaching life in terms of personal and social opportunities [5,6].

For other authors [7,8,9,10,11], the empowerment construct includes personal attributes such as a sense of competence, influence, and self-efficacy, which drive behaviors aimed at achieving specific results and goals. Therefore, the empowerment process means overcoming a situation of powerlessness and gaining control over one’s own life through individual capabilities and resources [4], with the aim of enhancing self-determination, autonomy, decision-making, and quality of life, generally.

Based on this description, Deere and Leon (2002) [11] specify that empowerment is not a linear process, i.e., it does not have a clearly defined beginning and end and it is not identical for all individuals. Instead, empowerment is experienced in a different, somewhat unique way by each person and develops in a distinct way according to personal history and context. It may thus result from various experiences, such as educational, organizational, work processes, etc. For example, empowerment has been studied in various disciplines to understand and promote the integration of disadvantaged or socially vulnerable groups, such as ethnic minorities [12], women [13] and the role of accounting in supporting family and people with reduced mobility [14].

Reduced mobility can be defined as the difference in a person’s functioning compared to most of the population when performing everyday tasks (moving, reading, holding, going to the bathroom, communicating, relating, etc.). The impact on quality of life depends on a large array of variables, including contextual factors, and on the type of reduced mobility in question [15].

In relation to functional divergence and quality of life, a number of authors have focused their efforts on studying the influence of different constructs, such as empowerment.

For example, Suriá [3] analyzed the empowerment of university students with and without disabilities and its association with academic performance. The results reflected that students without disabilities were more greatly empowered compared to students with disabilities.

For comparative purposes, another study analyzed the degree of empowerment of a group of young people according to the type of reduced mobility and the stage at which the reduced mobility was acquired. High levels of empowerment were observed among young people, and to a greater extent in young people with acquired disabilities, as well as with motor and visual disabilities [15]. Based on the related literature, empowerment is connected to several theories that emphasize self-improvement, such as goal-oriented behaviors and achieving specific results. Examples include competency theory, which is grounded in foundational knowledge; theories focused on process and intervention evaluation; and resilience theory.

### 1.2. The Study of Empowerment and Resilience

There has been mounting interest in the resilience construct in the scientific community. The term is borrowed from the field of physics and is defined as the ability of a material or substance to return to its original shape [16]. Thus, in the context of psychology, the resilience construct refers essentially to an individual’s ability to face adversity or negative life experiences and to emerge stronger from them, leading them to develop social, academic and vocational skills, despite exposure to stress and serious difficulties [17].

The theory of resilience is related to the concept of empowerment because it focuses on a person’s potential and development. The relationship between being resilient and being empowered is established in Grottberg’s now classic formula of resilience [18]: I have (networks of belonging), I am (body–mind–spirit integration), and I can (I am powerful in the sense that I am able to face, to be, to enjoy, to resolve, to bond, to protect myself, to take care of, to work, to feel, etc.).

The capacity for resilience is made up of personal and environmental factors that help people to face and overcome the different obstacles emerging in their lives [16,17,18,19,20]. We can infer that resilience is a dynamic feature, composed of a series of social and intrapsychic processes that take place over time, giving rise to combinations of individual attributes and the person’s environment. The concept therefore refers to an interactive process in which several components intervene, notably Social and personal Competence, Acceptance of Self of Life, and Self-Discipline [2,19,21].

In this sense, empirical evidence suggests that resilience is positively associated with the individual’s correct functioning in childhood [18], in adolescence [17], as well as in adulthood [2]. Nevertheless, these relationships become more complex when considering the different dimensions that make up resilience. For example, different authors have analyzed the relationship between this construct and the proper functioning of people across various personal domains, such as subjective well-being [3], optimism [22], and happiness [23]. Although positive relationships have been found between some resilience dimensions, such as Acceptance of Self and of Life and Optimism, or between Social Competence and Social Skills, the relationship between Self-Discipline and these domains is not so clear [24]. This fact suggests that not all the dimensions that make up resilience act in the same way on an individual’s well-being. It thus appears that differential resilience patterns must be considered when analyzing the relationship with other variables associated with quality of life.

Based on the concept of resilience and focusing on empowerment, similarities with the resilience dimensions mentioned above can be found in several empowerment components such as Self-Esteem-Self-Efficacy, Community Activism-Autonomy, Appropriate Anger, and lastly, Optimism-Control over the Future. A direct link may therefore exist between empowerment and resilience. In turn, the fact that both constructs are composed of different factors could imply that the resilience dimensions do not share the same significance and, thus, that each dimension has a different weight in the development of empowerment.

In relation to people with reduced mobility, different authors emphasize the relationship between empowerment and resilience [8,25,26,27]. The literature on the subject, however, has focused mainly on people with mental diversity [27,28], overlooking the issue of empowerment of people with other types of reduced mobility, such as reduced mobility. Moreover, focusing on resilience in adults with reduced mobility, to the best of our knowledge, no studies have hitherto been conducted on the possible relationship between different profiles of resilience and empowerment. Finally, no study seems to have been published either on the different levels of empowerment found in the distinct resilience profiles of adults with reduced mobility, addressing not only the empowerment construct as a whole, but also each empowerment sub-dimension (e.g., Self-Esteem-Self-Efficacy, Power/Powerlessness, Community Activism-Autonomy, Optimism-Control over the Future, and Appropriate Anger).

Based on all the above, the aim of the study was to further explore the relationship between empowerment and resilience in adults with reduced mobility. To this end, two objectives were proposed:

The first was to identify in a sample of people with reduced mobility whether there were any possible combinations of different resilience dimensions that gave rise to different profiles depending on the weight of each resilience dimension, Social and personal Competence, Acceptance of Self of Life, and Self-Discipline. Subsequently, we analyzed whether the resilience profiles obtained presented any statistically significant differences in the various empowerment factors.

In the second objective, an analysis is conducted to determine whether there are statistically significant differences between the resilience profiles obtained across the different factors that make up empowerment (Self-esteem–Self-efficacy, Power–Powerlessness, Community activism–Autonomy, Optimism–Control over the future, and Appropriate anger).

Based on these objectives, we expected that:

**H1.**  *Different groups of people with reduced mobility are expected to be found, with different resilience profiles depending on the weight of each resilience dimension.*

**H2.**  *Statistically significant differences in empowerment will exist based on the resilience profiles obtained. Specifically, the group with high resilience will score high on the empowerment dimensions.*

## 2. Materials and Methods

### 2.1. Participants

For reasons of access, the sample was purposive. It consisted of 94 people with reduced mobility belonging to the ASPAYM association (Association of Paraplegics and People with Great Physical Disabilities) in the Valencian Community, a Spanish association with 900 members. The eligible population was initially made up of the 142 members aged over 18 years who attended one of the meetings periodically convened by the association in Alicante (Spain). Of these, 51% were female and 49% male, aged between 21 and 62 years (M = 29.35; SD= 6.43). Regarding education levels, 44.00% had primary education, 30.00% secondary education, 17.20% university education and 8.80% indicated that they had no education. In terms of their labor force status, 37.90% indicated that they were neither studying nor working at the time, 35.40% that they were in active employment, 15.30% that they were studying, and 11.30% performed housework.

To check that there were no statistically significant differences between the participants, we used the Chi-square test for homogeneity of the frequency distribution, sex x age (χ^2^ = 10.09; *p* = 0.17).

### 2.2. Procedure

The data collection procedure consisted of administering the scales to the participant sample. These were administered to 94 members of the association. The latter had expressed their wish to collaborate after attending presential meetings in which the researcher, linked to the association, had explained the study objective. The questionnaires used to collect the information were administered in person at the same meetings once the participants had given their consent in writing. The scales were administered adapting to the participant’s conditions and the completion time was approximately 20 min. The data was collected between January and September 2024.

This study complied with the Declaration of Helsinki (2000) and the data protection regulations established in Royal Decree 1720/2007 implementing Organic Law 15/1999, ensuring the protection of participants’ privacy and confidentiality throughout all phases of the process. Ethical approval was requested and obtained from the Ethics Committee of the University of Alicante, in accordance with institutional requirements.

### 2.3. Instruments

- An ad hoc sociodemographic questionnaire to collect the sociodemographic data: sex, age, level of education, and employment status.
- The Resilience Scale was designed by Wagnild and Young [29] and validated in Spanish by Heileman et al. [30]. This scale consists of three distinct dimensions: 1. Social and Personal competence, related to beliefs in personal ability to achieve success, active coping, perseverance, social skills, assertiveness, etc. 2. Acceptance of self and life, which relates to adapting to situations, emotional balance, positive feelings in the face of difficult circumstances, etc. 3. Self-discipline, characterized by self-control, perseverance, emotional intelligence, willpower, etc. The instrument comprises 25 items using a seven-point Likert scale (from 1 = “strongly disagree” to 7 = “strongly agree”). Higher scores indicate greater resilience, with a scoring range from 25 to 175 points.

Regarding the psychometric properties of the instrument, these are adequate both in the original adaptation and in the Spanish version [31,32,33], showing reliabilities of 89% and 93%, respectively. The total variance explained by the latter version was 82.60% (35.40% for Acceptance of Self and Life, 23.40% for Social Competence, and 22.8% for Self-Discipline). In relation to the present study, adequate internal consistency was obtained through Cronbach’s alpha coefficient (α = 0.87).

- “Empowerment scale”, developed by Rogers et al. [34], is designed to measure the level of this potential capacity. It was translated into Spanish [3]. The scale has a total of 28 items, answered on a 4-point Likert scale (1 = strongly disagree, 4 = strongly agree). The information requested includes aspects related to the subject’s self-perceived decision-making capacity.

Additionally, the scale assesses four key factors that make up empowerment: self-esteem and self-efficacy, which reflect confidence and a positive appraisal of one’s own abilities; community activism and autonomy, indicating active participation in the community and the ability to act independently; appropriate anger, referring to the ability to express dissatisfaction in an adequate and constructive manner; and finally, optimism and control over the future, encompassing hope and the perception of control over upcoming events.

The maximum score is 112 points, but the score was divided into three ranges based on thresholds in the following way: low level = from 29 to 56; medium level = from 57 to 84; and high level = from 85 to 112.

This scale was chosen according to several criteria: because its application is brief; because it is validated for the young and adult population; and finally, because of the psychometric properties shown in the original version [34]. Thus, a reliability of 86% (α = 0.86) and an explained variance of 53.9% were found for the original scale, defined by five factors: Factor 1. Self-esteem-Self-efficacy (explains 24.5% of the total variance; Factor 2. Power/Powerlessness (12.4% of total variance explained); Factor 3. Community Activism-Autonomy (explains 7.6% of the total variance); Factor 4. Optimism-Control over the future (explains 5.4% of the total variance); Factor 5. Appropriate anger (explains 4% of the total variance).

To verify whether the scale was suitable for this study, Reliability was tested using Cronbach’s alpha, which indicated a reliability of 82.00% (α = 0.82).

### 2.4. Data Analysis

To identify resilience profiles, cluster analysis techniques were applied using two complementary methods. First, a hierarchical analysis was performed using Ward’s linkage method and squared Euclidean distance as the similarity measure, which allowed exploring the data structure without needing to predefine the number of clusters.

Subsequently, the K-means algorithm was applied, which assigns each individual to the cluster with the nearest mean, optimizing the partition. To determine the optimal number of clusters in the K-means analysis, the silhouette index was used, which quantifies internal cohesion and separation between groups, helping to validate the quality of the clustering.

To assess the consistency between the solutions obtained by both methods, the Adjusted Rand Index (ARI) was calculated, which measures the agreement in individual assignments to clusters, correcting for chance agreement. Values close to 1 indicate high concordance and stability between methods.

After establishing the different groups through cluster analysis, analyses of covariance (ANCOVA) were performed to examine the statistical significance of the differences between the groups in the empowerment dimensions, controlling for the potential influence of age, educational level, and employment status. The η^2^ index was applied to assess the magnitude or effect size of these differences.

Finally, for the analyses in which comparisons were statistically significant, post hoc tests were conducted to identify the groups that presented the differences. The Scheffé method was followed because the number of participants varied in each group and this test does not require the sample sizes to be equal. We also calculated the effect size (standard mean difference or d-index [35] to calculate the magnitude of the observed differences. Data were analyzed using the SPSS statistical package version 19.0.

## 3. Results

### 3.1. Combinations of Different Resilience Dimensions

**Figure 1:**
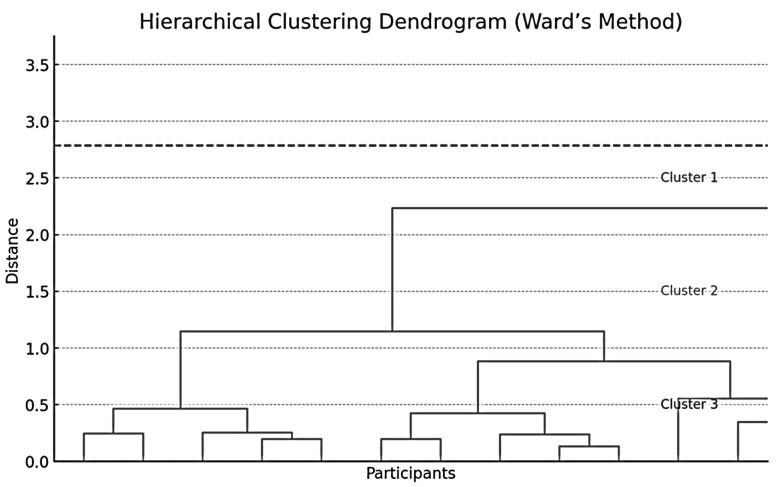
Dendrogram of Hierarchical Analysis (Ward’s Method with Squared Euclidean Distance).

The hierarchical analysis identified three clusters as the most parsimonious and coherent solution according to the dendrogram (see Figure 1).

Validation with K-means (Quick Cluster Analysis) and the silhouette index confirmed that the three-cluster solution provided the best separation and internal homogeneity (mean silhouette value >0.50). Case assignment from the hierarchical method largely coincided with the K-means partition (>90% agreement), reinforcing the stability of the solution.

**Figure 2:**
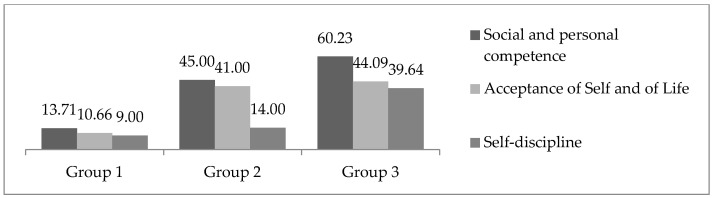
Graphical representation of the three-cluster model: Cluster 1 (Low Resilience), Cluster 2 (High Social and personal Competence, High Acceptance and Low Self-Discipline), Cluster 3 (High Resilience).

Both approaches identified an optimal structure composed of three clearly differentiated clusters. Cluster 1 (*n* = 26; 27.65%) showed low scores across the three dimensions of the Resilience Scale (Wagnild & Young, 1993) [29]: Personal Competence, Self- and Life-Acceptance, and Self-Discipline. Cluster 2 (*n* = 32; 34.04%) exhibited high scores in Personal Competence and Self- and Life-Acceptance, but low scores in Self-Discipline. Finally, Cluster 3 (*n* = 36; 38.29%) was characterized by high scores across all three dimensions (see Figure 2).

To assess consistency between the two methods, the adjusted Rand index was calculated, yielding a high value (ARI = 0.89), indicating strong agreement in assigning participants to the same groups. This methodological convergence increases confidence in the stability and reliability of the final solution.

### 3.2. Resilience Profile Inter-Group Differences Regarding Empowerment

As can be observed in Table 1, participants generally presented average levels of empowerment in Group 2 (*M* = 57.88, *SD* = 14.82) and Group 3 (*M* = 59.98, *SD* = 16.59), while a low empowerment level was found in Group 1 (*M* = 54.02, S*D* = 13.38). Regarding the mean scores of the global scale, statistically significant differences were observed in the three clusters (*F*_(2, 88)_ = 7.38, *p* < 0.001, *η^2^* = 0.54), and Group 3 presented higher means than Group 2 (*d* = 0.58) and Group 1 (*d* = 0.84). Group 2 also obtained higher mean scores than Group 1 (*d* = 0.61).

With respect to the factors composing empowerment, statistically significant differences were found for four of the five empowerment factors, with the exception of self-efficacy–self-esteem. Before conducting post hoc comparisons, ANCOVAs were performed to control for the potential influence of age, educational level, and employment status. These covariates did not show statistically significant effects on any of the empowerment factors (all *p* > 0.05), indicating that the observed differences between clusters were not attributable to these sociodemographic variables.

We then examined the empowerment factor scores that presented differences and the post hoc comparisons to determine the groups between which the differences existed. We found that statistically significant differences existed between the clusters with respect to Factor 2, *Power/Powerlessness* (*F*_(2, 88)_ = 3.78, *p* < 0.05, *η^2^* = 0.28), with the group scoring high on the three resilience dimensions (Group 3) obtaining higher scores than Group 2 as well as higher scores than the group with low scores in these dimensions (Group 1) for this factor. Thus, Group 3 had higher mean scores than Group 2 (*d* = 0.44) and higher mean scores than Group 1 (*d* = 0.52).

In relation to Factor 3, *Community Activism*, Group 3 presented higher scores than Group 2 (*F*_(2, 88)_ = 17.40, *p* < 0.001, *η^2^* = 0.39, *d* = 0.46) and higher scores than Group 1 (*d* = 0.52). Similarly, Group 2 showed higher means than Group 1 (*d* = 0.39).

With regard to Factor 4, *Optimism–Control over the Future*, statistically significant differences were also observed between the three clusters, with Group 3 notably presenting mean scores above those of Group 1 (*F*_(2, 88)_ = 9.22, *p* < 0.001, *η^2^* = 0.42, *d* = 0.53) and of Group 2 (*d* = 0.40).

A similar trend was found in the post hoc analysis regarding Factor 5, “Appropriate anger”, in which statistically significant differences were observed between the clusters (*F*_(2, 88)_ = 3.86, *p* < 0.05, *η^2^* = 0.21). Thus, the group that reported high scores on the three resilience dimensions (Group 3) showed higher scores than Group 2 and then the group with low scores on these dimensions (Group 1). The effect size of these differences ranged from (*d* = 0.34 to 0.39).

## 4. Discussion

In this paper, we explored the relationship between resilience and empowerment in people with reduced mobility. To this end, different objectives were proposed. The first was to analyze the possible combinations of the participants’ resilient dimensions to determine the weight of these resilient profiles in the development of empowerment.

Regarding the *first objective*, distinct resilience profiles were identified, each characterized by different combinations of resilience dimensions. Through a cluster analysis, three resilience profiles emerged, confirming the first hypothesis. These profiles included:

Group 1, consisting of individuals with low scores across all three resilience components. Group 2, characterized by high scores in Social and Personal Competence and Acceptance of Life and Self, but low scores in Self-Discipline. Group 3, which exhibited high scores in all three resilience dimensions, namely Social and Personal Competence, Self-Discipline, and Acceptance of Self and Life. These findings validate the first hypothesis of the study.

From these results, several key observations can be made. First, Group 1 represents individuals with disabilities who demonstrate low resilience levels across all dimensions. This profile is associated with poorer psychological adjustment and overall lower quality of life, reinforcing the notion that not all individuals with reduced mobility adapt positively to living with a reduced mobility [2]. Second, Group 2 presents a distinct resilience pattern, with notable strengths in Social and Personal Competence, as well as Acceptance of Self and Life, but low levels of Self-Discipline. These results suggest that not all dimensions of resilience contribute equally to the development of resilient capacity [24,28]. Finally, Group 3, as well as Group 2 (which displayed high scores in Social and Personal Competence and Acceptance of Self and Life), supports previous theories suggesting that living with reduced mobility can foster personal strength and enhance coping abilities [1]. This aligns with findings from other studies indicating that individuals with reduced mobility often exhibit high levels of resilience [8,9].

Regarding the *second objective*, the findings support the second hypothesis, which proposed that statistically significant differences in empowerment would be observed among the identified clusters. The results confirmed this hypothesis, as four out of the five empowerment factors showed significant differences, with Self-Efficacy/Self-Esteem being the only exception.

These findings contribute to validating the existence of distinct resilience profiles while also enhancing the understanding of the relationship between resilience and empowerment. Overall, participants demonstrated moderate levels of empowerment. This aligns with previous research suggesting that individuals with reduced mobility, particularly those with reduced mobility, often develop a sense of control over their lives and exhibit relatively indifferent reactions to stigma [26,36].

However, it is important to note that these findings do not suggest that individuals with disabilities do not experience negative emotions. Rather, they indicate that positive and negative emotions coexist in challenging circumstances, with negative emotions potentially serving as a catalyst for developing coping mechanisms and adapting effectively [36].

When examining the resilience-based groups and their average empowerment levels, it is particularly noteworthy that Group 3, which scored high across all three resilience dimensions, also achieved the highest empowerment scores across most factors. This finding aligns with existing research suggesting that resilience is shaped by a variety of personal and contextual variables and tends to develop through experiences of adversity—such as living with a reduced mobility [2,14,15]. Thus, it is reasonable to expect a connection between the components of resilience and the dimensions of empowerment [3].

Further support for this relationship can be found in the distribution of participants: Group 3 (high resilience) included more individuals than the groups with lower resilience, indicating that a generalized resilient profile may be more common than a low-resilience one within the studied population.

Additionally, the strength of the association between empowerment and resilience is underscored by the effect sizes observed. Group 3 consistently outperformed Group 1 (low resilience) on empowerment measures, with notably large effect sizes in several key areas: Factor 2: Power/Powerlessness, Factor 3: Community Activism, Factor 4: Optimism and Future Control and Factor 5: Appropriate Expression of Anger

These results indicate that individuals with higher levels of Social Competence, Acceptance of Self and Life, and Self-Discipline—the three core dimensions of resilience—are also more likely to exhibit higher empowerment in these domains.

Several scholars [37,38] have pointed out that empowerment is driven by behaviors aimed at achieving specific personal or collective goals. In this context, the resilience dimensions appear to function as internal enablers—skills and attitudes that promote a stronger sense of agency and goal-directed action.

Along the same lines, Zimmerman [39] points out that the intrapersonal dimension of empowerment comprises attributes of the self as a sense of social and personal competence. Thus, the resilient dimension of Social and Personal Competence would encompass behaviors necessary for interpersonal relationships, such as expressing feelings, attitudes, desires, opinions, or rights in a way that is appropriate to the situation, respecting those behaviors in others [37]. In turn, Personal Competence embraces aspects related to a person’s capacity, mastery, perseverance, and ability, among other constructs [40]. This is most reflected in the empowering factors related to power/powerlessness, optimism/control over the future, and appropriate anger.

On the other hand, Factor 1: Self-Esteem/Self-Efficacy did not show significant differences between the resilience groups. One possible explanation is the persistent influence of societal beauty standards and stereotypes, which may negatively affect the self-perception of individuals with disabilities [24]. This could account for consistently lower scores on self-esteem-related items across all groups, regardless of resilience level.

Taken together, the findings support the notion that resilience and empowerment are closely interlinked, with Acceptance of Self and Life and Social Competence appearing to be the most influential resilience components in fostering empowerment.

### Limitations and Future Directions

Despite these valuable insights, the study has several limitations. It is important to emphasize that, given the cross-sectional design of this study, it is not possible to establish causal relationships between resilience and empowerment. The observed association could be bidirectional, where both constructs influence each other, or mediated by variables not considered in this analysis.

Therefore, it is recommended that future research adopt longitudinal or intervention-based designs to examine the direction and causal nature of this relationship. This approach would provide a deeper and more robust understanding of the link between resilience and empowerment, promoting the development of more effective strategies to support people with reduced mobility.

Regarding the sample, although the small size limits the generalizability of the results, the specific selection of participants with motor disabilities ensures the relevance of the findings for this population. However, accessing a larger group of participants with reduced mobility can be challenging, so the promising nature of these results suggests that further research with expanded samples would be worthwhile.

Although all participants have a reduced mobility degree of 65% or higher, there may be functional variability between individuals with paraplegia and those with tetraplegia, which could influence their levels of resilience and empowerment. This variability includes differences in physical capabilities, independence in daily activities, and the extent of support required, all of which can affect psychological and social outcomes. The relative homogeneity of the sample in terms of overall reduced mobility level limits the ability to analyze these differences in depth. Therefore, future studies should consider stratifying participants by specific types and severities of motor impairment to better understand how these factors impact resilience and empowerment. Such an approach would provide more nuanced insights into the diverse experiences within the population of people with reduced mobility and help tailor interventions more effectively.

Another limitation related to the sample in this study lies in the low educational level observed. In the Spanish context, until relatively recently, educational and social integration policies did not effectively encourage people with disabilities to continue their studies beyond compulsory education. This historical factor has contributed to lower educational attainment in part of this population, which partly explains the sociodemographic composition of the sample. This aspect should be considered when interpreting the results, as it may influence the generalizability of the findings. Nevertheless, we acknowledge that to enhance external validity and the applicability of the conclusions to other cultural, educational, or socioeconomic contexts, future research should adopt multisite sampling strategies and include a greater diversity of participants.

Additionally, the use of a self-selected sample—participants voluntarily agreed to complete the questionnaire—may have introduced bias. Those who chose to participate might have had different motivations or expectations than those who did not. Future research should aim to control for self-selection effects to improve internal validity.

It would also be beneficial to complement this quantitative approach with qualitative methods. This would allow researchers to gain a more nuanced understanding of how resilience influences empowerment from the perspective of individuals living with reduced mobility.

## 5. Conclusions and Implications

Despite these limitations, the findings of this study contribute significantly to a deeper understanding of the complex relationship between resilience and empowerment. The results indicate that fostering specific resilience profiles could play a crucial role in enhancing empowerment, particularly among individuals with reduced mobility. By identifying the key characteristics and factors that support resilience in this population, this study opens new avenues for tailored interventions.

Moreover, these insights have practical implications for the development of policies, programs, and support services that aim not only to improve resilience but also to promote greater autonomy, participation, and overall quality of life. Strengthening resilience could serve as a foundational strategy in empowering individuals to better navigate daily challenges, reduce vulnerability, and increase their capacity to achieve personal goals.

To strengthen the practical applicability of the identified resilience profiles, concrete examples of training and empowerment programs aimed at individuals with reduced mobility were incorporated. For instance, implementing training programs focused on personal autonomy skills, which include workshops for developing competencies in managing daily life, thereby enhancing self-discipline and self-efficacy, is particularly useful for Cluster 1, characterized by low resilience scores.

Similarly, courses on acceptance and emotional well-being could be offered, aimed at fostering self- and life-acceptance through mindfulness techniques and cognitive-behavioral therapy, primarily targeting Cluster 2, which shows high personal competence but lower self-discipline.

Finally, it would be valuable to establish comprehensive empowerment and leadership programs that combine the development of social skills, promotion of autonomy, and community participation. These would support a global strengthening of the assessed dimensions and be especially suitable for Cluster 3, characterized by high scores across all resilience dimensions.

This segmented approach allows policies and programs aimed at people with reduced mobility to be more effective by focusing resources and strategies based on the specific psychosocial characteristics of each profile, which in turn promotes greater adherence and positive outcomes in terms of well-being and quality of life.

Although this study reports adequate internal consistency of the scales used to measure resilience and empowerment, measurement invariance and factorial validity were not specifically addressed in the particular context of individuals with motor disabilities. This limitation is important, as the validity and equivalence of scales may vary according to cultural, functional, or contextual characteristics of the sample. However, previous studies have shown that both the Resilience Scale (Wagnild & Young, 1993) [29] and empowerment measures have demonstrated satisfactory psychometric properties in populations with diverse disabilities, including individuals with reduced mobility [3]. These findings suggest that the scales employed maintain a good level of applicability and effectively capture relevant constructs within this group.

Nonetheless, future research should further explore measurement invariance to ensure that the scales function equivalently across specific subgroups within this population, thereby strengthening external validity and the interpretation of results. This line of inquiry would contribute to optimizing the precision of assessments and the effectiveness of interventions based on these constructs.

Future research should explore these dynamics further, ideally through longitudinal and culturally diverse studies, to validate and expand upon these findings. In the meantime, practitioners and policymakers are encouraged to integrate resilience-building strategies into their approaches, ensuring they address both psychological and structural barriers faced by people with reduced mobility.

## Figures and Tables

**Table 1:** Means and standard deviations obtained by the three groups and values of eta squared (η^2^) for each empowerment dimension.

Empowerment Factors	Group 1 Low Resilience		Group 2 High Accept. High Comp. Low Self-Disc.		Group 3 High Resilience		*F*	*p*	*η^2^*
	*M*	*SD*	*M*	*SD*	*M*	*SD*			
Factor 1. Self-Esteem-Self-Efficacy	15.82	4.92	15.92	3.59	16.06	4.49	4.06	0.64	0.09
Factor 2. Power/Powerlessness	16.06	2.80	16.16	2.72	17.11	2.84	3.78	0.037	0.28
Factor 3. Community Activism	9.98	3.81	10.77	2.69	12.21	3.32	17.40	0.000	0.39
Factor 4. Optimism-Control Future	4.21	1.34	4.26	1.15	6.32	1.26	9.22	0.000	0.42
Factor 5. Appropriate Anger	3.48	0.89	3.51	0.82	4.56	0.99	3.86	0.048	0.21
Total	54.02	13.38	57.88	14.82	59.98	16.59	7.38	0.000	0.54

## Data Availability

Where no new data were created, or where data is unavailable due to privacy or ethical restrictions.
